# Progressive crushing ^40^Ar/^39^Ar dating of a gold-bearing quartz vein from the Liaotun Carlin-type gold deposit, Guangxi, southern China

**DOI:** 10.1038/s41598-022-17061-x

**Published:** 2022-07-27

**Authors:** Rongguo Hu, Baocheng Pang, Xiujuan Bai, Fraukje M. Brouwer, Lingan Bai, Xijun Liu, Yuanqiang Li, Jianqi Xu, Huaning Qiu

**Affiliations:** 1 College of Earth Sciences and Guangxi Key Laboratory of Hidden Metallic Ore Deposits Exploration, Guilin University of Technology, Guilin, 541004 Guangxi China; 2 Key Laboratory of Tectonics and Petroleum Resources of Ministry of Education, China University of Geosciences (Wuhan), Wuhan, 430074 China; 3 Department of Earth Sciences, Faculty of Science, VU Amsterdam, De Boelelaan 1085, 1081 HV Amsterdam, The Netherlands

**Keywords:** Geochemistry, Economic geology, Geochemistry

## Abstract

Carlin-type gold deposits are among the largest hydrothermal gold deposits in the world. However, direct dating the metallogenic age of these deposits is difficult, because not all deposits provide material suitable for conventional radiometric methods. Syn-mineralization stage quartz veins from these deposits usually contain abundant fluid inclusions, which allow fluid inclusion ^40^Ar/^39^Ar dating. In this study, progressive crushing ^40^Ar/^39^Ar dating has been performed on a gold-bearing quartz vein from the Liaotun Carlin-type gold deposit in northwestern Guangxi, China. Argon isotopes liberated from the later steps yielded an isochron age of 200.7 ± 2.1 Ma. We infer that Ar-bearing gas was extracted from the primary fluid inclusions, and that the age of ca. 200.7 Ma reflects the timing of gold mineralization. The initial ^40^Ar/^36^Ar ratio corresponding to the isochron is 298.0 ± 4.3, which is statistically indistinguishable from the value for air, indicating that the ore-forming fluids probably mainly derived from gravitational pressure flow in the basin of air-saturated water. Our preliminary study shows the feasibility and great potential of ^40^Ar/^39^Ar dating of gases from fluid inclusions by progressive crushing of quartz veins to date the mineralization age and decipher the fluid origins of Carlin-type gold deposits.

## Introduction

**Figure 1:**
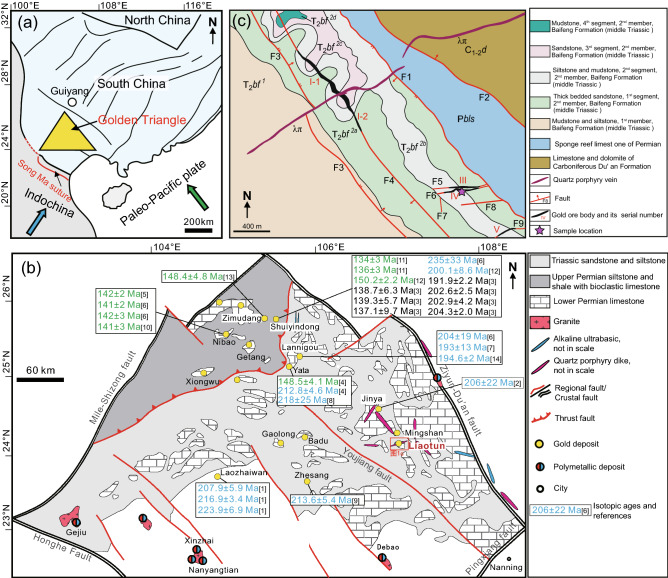
(**a**) Simplified geological map of the tectonic framework and location of the Golden Triangle; (**b**) Regional geological sketch map showing the distribution of gold deposits representative isotopic ages in and around the Golden Triangle and the location of the study area. (**c**) Geological map of the Liaotun gold deposit. This Figure modified after references^4^ and^18^ and was created using Adobe Illustrator version 2022 (https://www.adobe.com/products/illustrator.html), edited by Rongguo Hu. Age references: [1]-^2^; [2]-^6^; [3]-^4^; [4]-^19^; [5]-^20^; [6]-^21^; [7]-^22^; [8]-^8^; [9]-^23^; [10]-^15^; [11]-^24^; [12]-^25^; [13]-^26^; [14]-^27^.

The Yunnan-Guizhou-Guangxi-area, or Dian-Qian-Gui-area, of southwestern China is known as the Golden Triangle since this region contains the second-largest concentration of Carlin-type gold deposits in the world (Fig. 1a,b), with total resources of > 800 tons of Au at an average grade of 4.5 g/t^1–10^. Precise age determinations of extremely fine disseminated mineralization, such as Carlin-type gold deposits are not always available, since they generally lack datable minerals for conventional isotopic dating techniques^5,11–14^. However, with the development and utilization of new techniques in mineral separation and isotopic analysis, great progress have been made in constraining the metallogenic ages of Carlin-type gold deposits during the past decades^4,14,15^. For example, the metallogenic ages of Carlin-type gold deposits in Nevada, USA, have been constrained at on 42–36 Ma by applying Rb–Sr and ^40^Ar/^39^Ar dating methods to galkhaite and adularia, respectively^11,16,17^.

In the Carlin-type gold deposits from the Dian-Qian-Gui area, no galkhaite or adularia has as yet been reported and therefore the published isotopic dating results from this region (Fig. 1b) are mainly derived from Rb–Sr dating of hydrothermally altered minerals and fluid inclusions^19–21,28^, arsenopyrite, pyrite and pyrobitumen Re-Os dating^6,8,22^, dating of hydrothermal rutile, monazite, calcite or apatite by the U-(Th)-Pb method^4,9,15,23^, zircon U-Th-He dating^29^, sericite and illite ^40^Ar/^39^Ar dating^27^, and Sm–Nd dating of hydrothermal calcite^24–26^. In summary, the reported geochronological data indicate that the Dian-Qian-Gui Golden Triangle region underwent two independent gold mineralization events during the late Triassic to early Jurassic (230–195 Ma) and the late Jurassic to early Cretaceous (150–122 Ma).

The Liaotun gold deposit in Bama County, northwest Guangxi (Fig. 1b,c), is a typical Carlin-type gold deposit in the Golden Triangle and the only one whose ore bodies are crosscut by Late Yanshanian quartz porphyry veins. Precisely constraining the mineralization age of this gold deposit will not only help to reveal the genetic link between these felsic dikes and ore-formation, but also contribute to further exploration of ore deposit. However, the mineralization age of the Liaotun gold deposit is poorly constrained mainly because it contains no suitable minerals for traditional isotopic dating methods. Quartz veins from these deposits that are coeval with the mineralization usually contain abundant K-rich fluid inclusions, allowing fluid inclusion ^40^Ar/^39^Ar dating.

Gold deposits in the Liaotun area are hosted by the middle Triassic Baifeng Formation (T_2_*bf*) that consists of mudstone, sandstone and siltstone, and the ore bodies are mainly controlled by NW-trending or EW-trending faults (Fig. 1c). A felsic dike intruded Carboniferous limestone and Triassic sandstone along an ENE- to NE-trending fault and cut the biggest orebody (No. I). ^40^Ar/^39^Ar dating of muscovite phenocrysts from this dike yielded a plateau age of 95.5 ± 0.7 Ma, which was interpreted as the lower limit of the metallogenic stage^18^. Later SIMS zircon U–Pb dating showed that the Liaotun dike was emplaced at 97.2 ± 1.1 Ma (MSWD = 2.9), and the authors inferred that there is no genetic link between the felsic dike and Liaotun Carlin-type gold deposit^30^. Given these inconclusive data regarding the precise mineralization age of this gold deposit, more accurate direct metallogenic data are needed.

The ^40^Ar/^39^Ar *in vacuo* progressive crushing technique for dating the ages of fluid inclusions has been improved and developed for thirty-five years^31,32^. This method has been widely applied to constrain the formation ages of hydrocarbon resources^33,34^, retrogression after high-ultrahigh pressure metamorphism^35,36^, and in particular, for direct dating of hydrothermal mineral deposits of cassiterite, sphalerite, and wolframite, and gangue minerals like mineralized quartz veins^32,37–43^. Nevertheless, this technique has not yet been successfully employed in sediment-hosted Carlin-type gold deposits, although mineralized quartz veins with abundant fluid inclusions are widely developed in this type of ore deposit.

**Figure 2:**
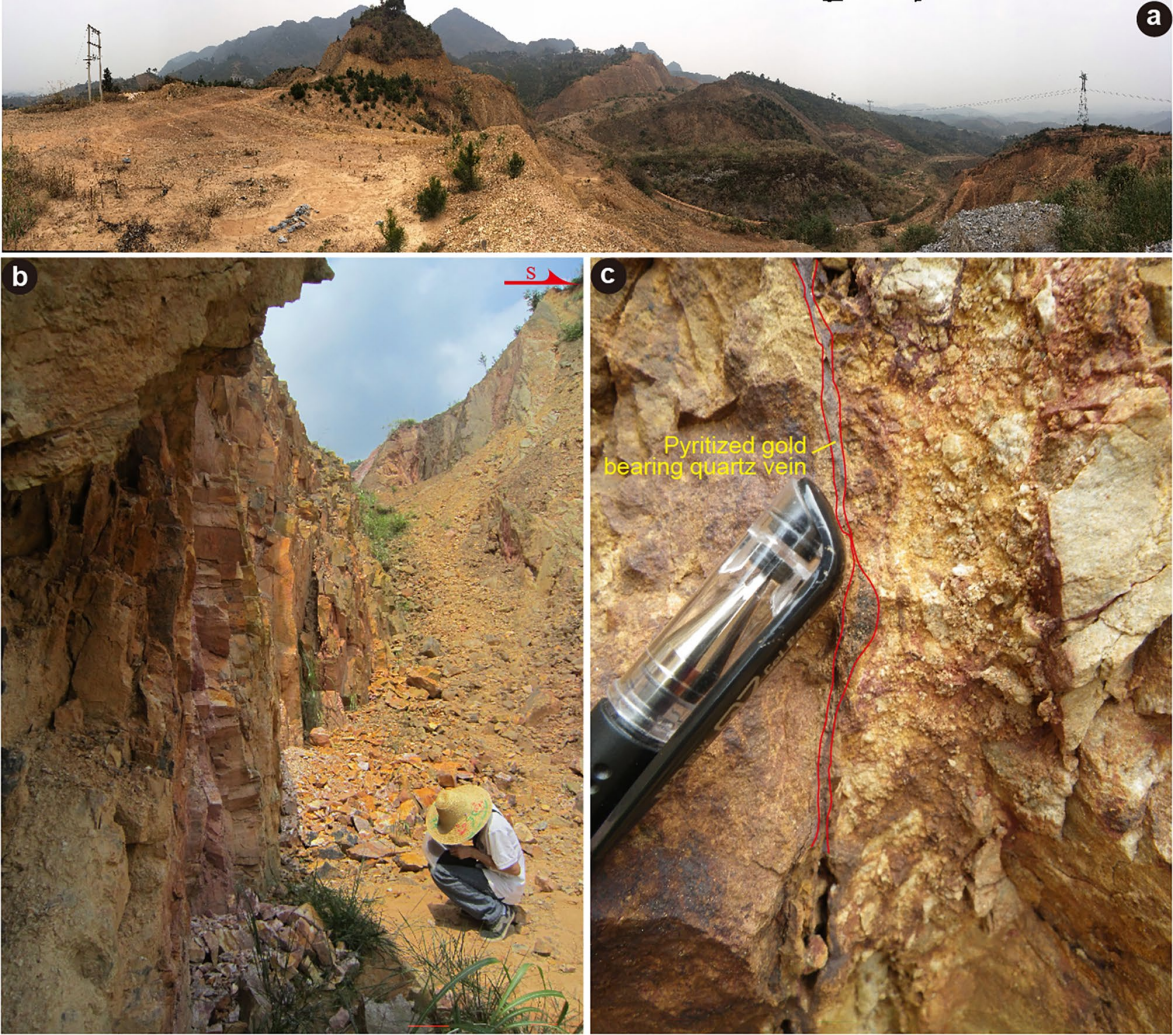
(**a**) Panoramic photo of Liaotun gold deposit, (**b**) goaf of ore body IV; (**c**) pyritization in an Au-bearing quartz vein in Permian quartz sandstone. Gold-bearing minerals include arsenian pyrite, arsenopyrite.

In this contribution, we apply the ^40^Ar/^39^Ar *in vacuo* progressive crushing dating technique to a pyritized gold-bearing quartz vein related to the main mineralization stage in the Liaotun Carlin-type gold deposit, northwest Guangxi (Fig. 2c). Based on a combined approach petrographic observation of fluid inclusions and micro-thermometric measurement, our study attempts to decipher the origin of fluid flow and to constrain the age of quartz vein formation by using a direct dating approach. Furthermore, our study demonstrates the feasibility of ^40^Ar/^39^Ar dating by *in vacuo* progressive crushing of quartz, resulting in the liberation of gas from fluid inclusions, and exploits this approach to constrain the mineralization age of the Carlin-type gold deposits, noting that such deposits typically lack minerals amenable for dating.

### Geological setting

The Dian-Qian-Gui ore deposits are restricted to the Devonian-Triassic Youjiang basin, which is bound to the northeast by the Ziyun-Du’an fault, to the northwest by the Mile-Shizong fault, and to the southeast by the Pingxiang fault, which separates the basin from the Cathaysia block (Fig. 1b)^1^. It contains widely developed Au-As-Sb-Hg low-temperature hydrothermal deposits and is one of the largest concentrations of Carlin-type gold deposits in the world^1–3,8,10^.

The evolution of the Youjiang Basin can be divided into six stages from Early Devonian to Cretaceous times^44^, while gold deposits in this region mainly formed during a postcollisional transpressional event in the Indochina orogen^1,2,4,6,44^. The gold deposits in the Golden Triangle are mainly hosted in Permian limestone and volcaniclastic sedimentary rocks or Triassic siliciclastic rocks and carbonates, and are structurally controlled by various folds and associated faults, likely produced during Indosinian orogenic deformation^1,45^.

### Geology of the Liaotun deposits

The fault-bound Liaotun gold deposit, in Bama County, Northwest Guangxi, is a medium-sized Carlin-type gold deposit, which is located on the southwestern margin of the isolated Longtian carbonate platform (Fig. 1b)^18,30,46^. The exposed sedimentary rocks in the platform are mainly limestone, intercalated with dolomite of the Carboniferous Du’an Formation (C_1-2_*d*) and Permian sponge reef limestone (P*bls*). The strata around the platform belong to the Triassic Baifeng Formation (T_2_*bf*), which consists of interbedded deep-water basin facies sandstone and mudstone (Fig. 2a)^18,30^.

The study area contains well-developed faults and linear folds, with individual gold orebodies structurally controlled by high-angle faults. Five NW-trending and four EW-trending faults have been recognized in the area (Fig. 1c). Among them, the NW-trending faults F1 and F2 are syn-sedimentary faults, while the NW-trending F4 and the EW-trending F5, F6, F9 faults are ore-bearing structures, hosting the ore bodies labelled I, III, IV, and V, respectively (Fig. 1c)^18,46^. Late Yanshanian (97–95 Ma) quartz porphyry veins intruded Carboniferous limestone and Triassic sandstone along an ENE- to NE-trending fault across the Longtian dome^18,30^.

The deposit consists of five orebodies and the largest orebody (No. I) is cut by the late Yanshanian quartz porphyry vein in the middle and the northwestern and southeastern parts have been labelled I-1 and I-2, respectively (Fig. 1c). Gold mineralization in the upper part of the orebody is oxidized ore, dominated by silicification and limonitization of detrital quartz greywacke and cataclasite. Primary to semi-primary ore minerals in the lower part of the orebody are disseminated pyrite and minor arsenopyrite. Hydrothermal alteration associated with gold mineralization in the deposit includes silicification, pyritization, arsenopyritization, (de)carbonation, clayization and sulfidization. The occurrence, textures, and mineral assemblages of the ores at Liaotun indicate that the hydrothermal alteration associated with gold mineralization in the deposit can be divided into four stages: (1) decarbonation + silicification stage; (2) quartz + pyrite + arsenopyrite stage; (3) quartz + stibnite stage; (4) quartz + calcite + clayization stage^18,46^.

The largest, NW-trending (F4) fault-controlled orebody I is 656 m long and on average 9 m thick, generally dips to SW with steep dip angles of 50° to 85° and has an average grade of 1.62 g/t Au. The smaller III and V ore bodies are controlled by EW-trending vertical F5 and F6 faults. Body III are 230 m long and 7.20 m thick with an average gold grade of 7.33 g/t, and orebody V is 194 m long, 1.16 m thick and has an average gold grade of 0.34 g/t Au^18,46^. Orebody V is hosted in siltstone, mudstone and thick-bedded sandstone in the second member of middle Triassic Baifeng Formation. In this orebody, the dominant ores are taupe and purplish red silicified fine-sandstone, cataclasite, crushed rock, minor silicified siltstone and bedded mud, and veinlet quartz usually can be observed locally (Fig. 1c). The ore structures are disseminated, spotted, micro-veined-network, brecciated, porous and earthy^18^. Sample LT19-1-2Qz, used in this study for fluid inclusion *in vacuo* crushing ^40^Ar/^39^Ar dating, was collected from the mine waste of orebody IV (Fig. 2b,c). It is a 0.5–2 cm wide pyritized gold-bearing quartz vein with a grade of 4.02 g/t Au^46^.

## Results

### Fluid inclusion analyses

**Figure 3:**
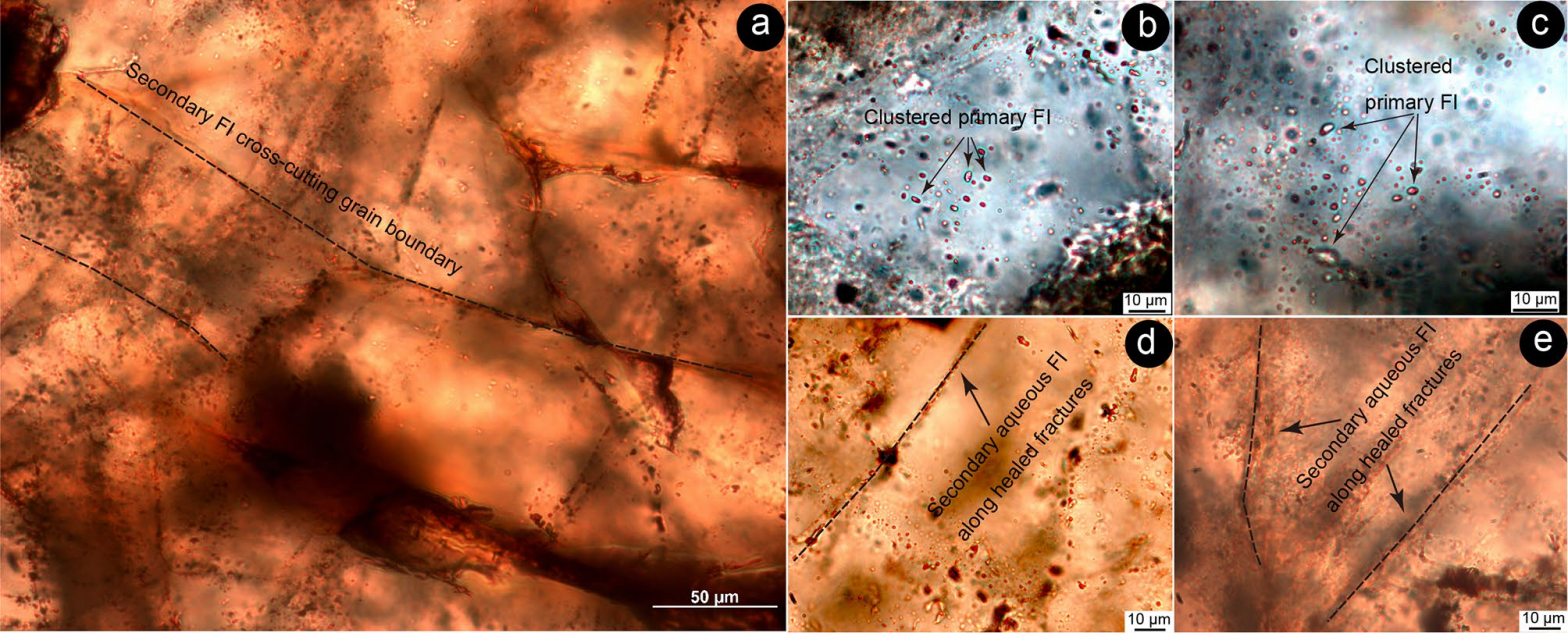
Microphotographs of fluid inclusions in sample LT19-1-2Qz from the Au-bearing quartz vein from Liaotun gold deposit. (**a**) primary and secondary fluid inclusions in vein quartz, and secondary fluid inclusions cross-cutting grain boundary; (**b**,**c**) isolated and clustered primary fluid inclusions in vein quartz; (**d**,**e**) secondary fluid inclusions in vein quartz along healed fractures.

Petrographic observation and micro-thermometric measurements have been applied to gold-bearing vein quartz sample LT19-1-2Qz from the Liaotun Carlin-type gold deposit. The total salinities (*W*) are calculated with the reduction formula based on the final ice-melting temperatures (|*T*_*m*_|):* W* = 1.78|*T*_*m*_| − 0.0442|*T*_*m*_|^2^ + 0.000557|*T*_*m*_|^47^. Fluid inclusions are, in places grouped in clusters (Fig. 3b,c). Two or three single fluid inclusions in each cluster were selected for measurement.

**Figure 4:**
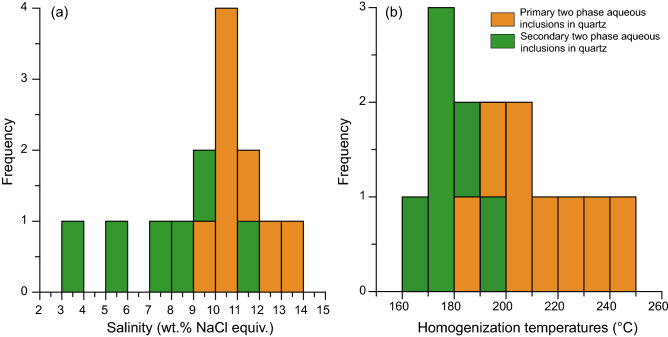
Histograms of salinities (**a**) and homogenization temperatures (**b**) of fluid inclusions in the Au-bearing quartz vein from the Liaotun gold deposit.

Abundant fluid inclusions developed in the vein quartz and can be separated into primary and secondary fluid inclusions (PFIs and SFIs, respectively) based on the textural criteria (Fig. 3a). Most of the PFIs are < 5 μm in diameter and characterized by two-phase, liquid–vapor contents with an extremely small H_2_O bubble at room temperature (Fig. 3b,c). They have negative crystal, round, elongate, or irregular shapes, and occur in isolated, random or clustered distributions (Fig. 3b,c), suggesting a primary origin. Heating-freezing stage analysis shows that the PFIs have T_m_ between − 6.5 and − 9.5 °C, corresponding to salinities of 9.9–13.4 wt.% NaCl equivalent (Fig. 4a). The homogenization temperature is between 245 and 180 °C (Fig. 4b). Tiny linear arrays of SFIs, ~ 1–3 μm long, mainly occur along cross-cutting healed fractures and have round, oval, tubular or irregular shapes (Fig. 3a,d,e), but some irregular SFIs reach 5-10 μm diameter. These inclusions are commonly pure aqueous inclusions, but two-phase, liquid–vapour inclusions occur locally. The secondary fluid inclusions yielded T_m_ values between − 2.1 and − 7.5 °C, corresponding to salinities of 3.5–11.1 wt.% NaCl equivalent (Fig. 4a). Values for T_h_ were between 200 and 160 °C (Fig. 4b).

### ^40^Ar/^39^Ar dating result

**Figure 5:**
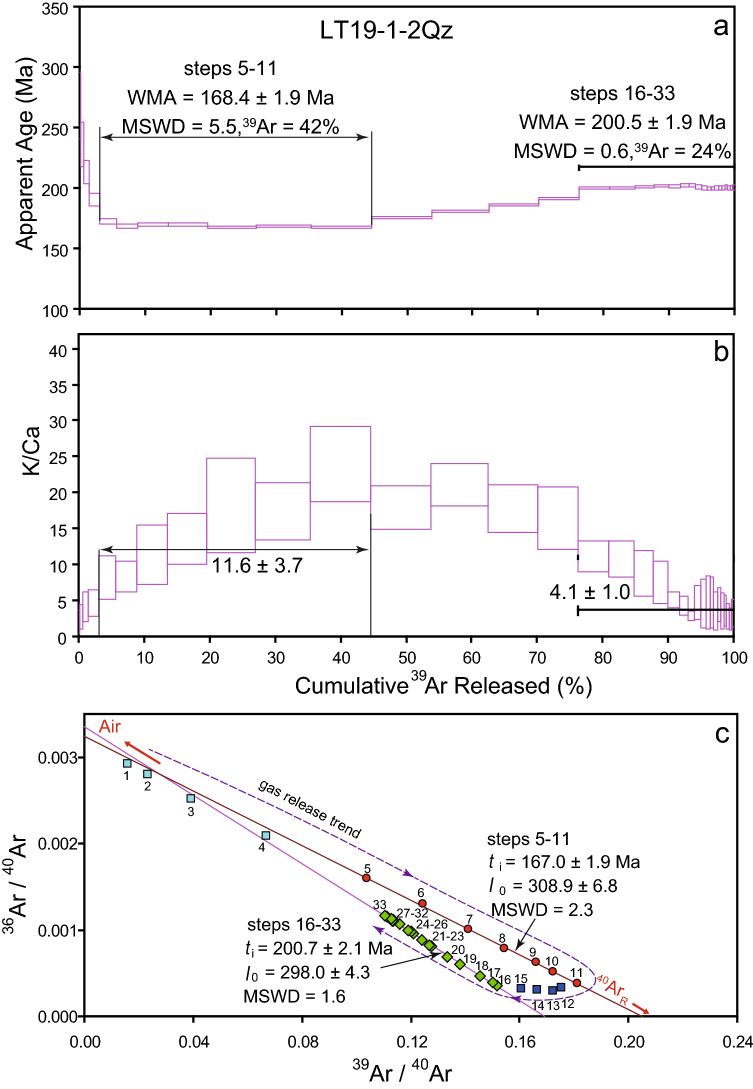
Plots based on the ^40^Ar/^39^Ar data of the quartz vein from the Liaotun Carlin-type gold deposit by *in vacuo* progressive crushing. (**a**) Age spectrum; (**b**) K/Ca spectrum; (**c**) inverse isochron. Data points through crushing (marked as 1 to 33) yield a clockwise trend, showing that radiogenic (^40^Ar_R_) and trapped argon in fluid inclusions and atmospheric argon (Air) from the crusher successively contribute to different parts of the degassing.

During the *in vacuo* crushing experiment, quartz separate LT19-1-2Qz was crushed in 33 stages with a total number of around 16,990 pestle drops (Appendix S1). The pestle drop number is increased step-by-step from tens in the first step to hundreds. The age spectra for this sample, shown in Fig. 4, yield a gradually decreasing staircase-shaped age spectrum with apparent ages from 268 to 191 Ma in the first four crushing stages. Subsequently, the apparent ages from stages 5 to 11 form a plateau, with a weighted mean age (WMA) of 168.4 ± 1.9 Ma (Fig. 5a, 2σ error, ^39^Ar = 42%, MSWD = 5.5) and an average K/Ca ratio of 11.6 ± 3.7 (Fig. 5b, 2σ). The steps defining the WMA yield an isochron with an age of 167.0 ± 1.9 Ma (2σ, MSWD = 2.3), corresponding to an initial ^40^Ar/^36^Ar ratio of 308.9 ± 6.8 (2σ, Fig. 5c). Apparent ages climb from 175.7 Ma for step 12 to 191.5 Ma for step 15, which is followed by a plateau defined by steps 16 to 33 with a weighted mean age of 200.5 ± 1.9 Ma (Fig. 5a, 2σ, ^39^Ar = 24%, MSWD = 0.6) and an average K/Ca ratio of 4.1 ± 1.0 (Fig. 5b, 2σ). On the inverse isochron diagram of ^36^Ar/^40^Ar *vs.* ^39^Ar/^40^Ar (Fig. 4c), these data points define an excellent linear array, and yield an isochron age of 200.7 ± 2. Ma (2σ, MSWD = 1.6) with an initial ^40^Ar/^36^Ar ratio of 298.0 ± 4.3 (2σ), which are consistent with the plateau age, as well as the atmospheric value for the ^40^Ar/^36^Ar ratio.

**Figure 6:**
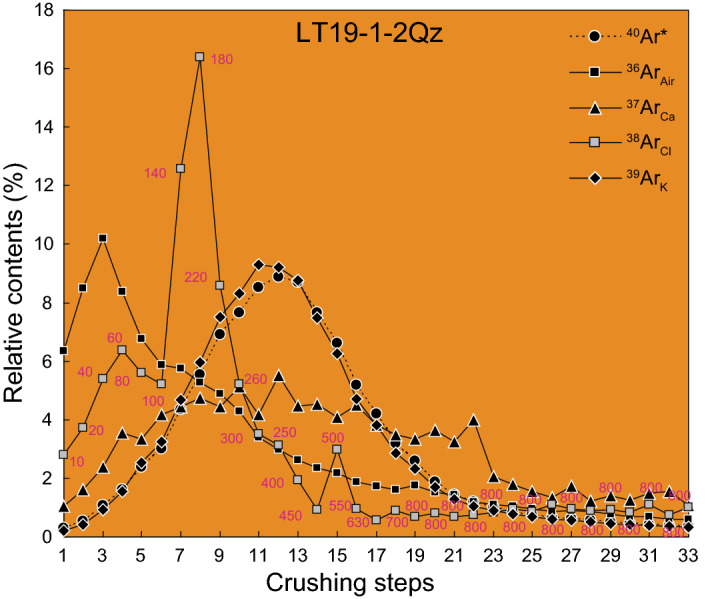
Release patterns of argon isotopes during the *in vacuo* progressive crushing experiment. The number of pestle drops per stage is marked on the ^38^Ar_Cl_ curve.

Five argon isotopes exist in ^40^Ar/^39^Ar analyses: ^36^Ar, ^37^Ar, ^38^Ar, ^39^Ar and ^40^Ar. In this study, all the argon isotopes are routinely applied interference corrections for the interfering nuclear reactions with isotopes of Ca, K, Ar and Cl. ^36^Ar_air_–atmospheric ^36^Ar; ^38^Ar_Cl_–produced by chlorine during irradiation after the air correction; ^39^Ar_K_–produced in the key reaction on ^39^ K during irradiation; ^40^Ar^⁎^–after air correction, including the radiogenic ^40^Ar from in situ decay of ^40^ K and the parentless excess ^40^Ar. Patterns of ^36^Ar_air_, ^37^Ar_Ca_, ^38^Ar_Cl_, ^39^Ar_K_ and ^40^Ar^⁎^ release for the quartz are presented in Fig. 6. The argon release patterns of sample LT19-1-2Qz by crushing indicate that very large amounts of ^38^Ar_Cl_ was released in the early crushing steps. Meanwhile, ^40^Ar^⁎^, ^39^Ar_K_ and ^37^Ar_Ca_ signals generally increase stepwise from very low signals at first, and subsequently with peaks at the middle crushing steps. The atmospheric ^36^Ar_Air_ signal gradually rises in the first three steps and then steadily decreases with continued crushing.

## Discussion

### Origin and evolution of the metallogenetic fluids

Detailed fluid inclusion studies have been done at the Shuiyindong, Lannigou, Yata, Taipingdong, Zimudang, Nibao, Mingshang and Liaotun in the Dian-Qian-Gui region^1^. As illustrated in Appendix table S2, two-phase aqueous fluid inclusions in early-stage quartz, main-stage quartz and late-stage quartz, calcite, fluorite and stibnite from Dian-Qian-Gui region deposits have T_h_ of 301–159 °C, 358–122 °C and 226–80 °C, corresponding to salinities of 0.7–13.7, 0.3–13.7, and 0.2–8.7wt.% NaCl equivalent, respectively. The data for the main-stage Au-bearing quartz vein from our fluid inclusions analysis are generally consistent with previous studies.

Reported noble gas (He, Ne, Ar) isotope data of fluid inclusions extracted from arsenopyrite, quartz, calcite and fluorite from Shuiyindong, Nibao and Yata Carlin-type gold deposits in the Golden Triangle indicate that the main ore-forming fluids were a mixture of ascending magmatic fluid and sedimentary pore fluid, whereas the late metallogenetic fluids were a mixture of sedimentary pore fluid or deeply sourced metamorphic fluid and shallow meteoric groundwater^1,3,20,45^. Moreover, in situ SIMS analysis on Au-bearing pyrite from the Jinya deposit, a Carlin-type gold deposit close to Liaotun (Fig. 1c), yields δ^34^S values (*ca.* − 6.22‰) similar to pyrite in the surrounding sedimentary basin (*ca.* − 7‰), suggesting that the fluids that formed the Jinya deposit may be meteoric waters transported by regional faults derived from the surrounding sedimentary basin^51^. Recently, Jin and co-workers^52^ reported crush-leach analysed solute data of fluid inclusion extracts from quartz, calcite, realgar, and fluorite from the Shuiyindong, Nibao, and Yata gold deposits in the Golden Triangle, and the results also suggest that the ore-forming fluids contain mixtures of basinal and magmatic-hydrothermal fluids.

The initial ^40^Ar/^36^Ar ratio of fluid inclusions provides constraints on the origin of the fluid^32,41^. Previous studies have demonstrated that deep magmatic metallogenic hydrothermal fluids, especially mantle derived hydrothermal fluids, generally contain excess ^40^Ar^32,40,41,43,53^. The initial ratios of ^40^Ar/^36^Ar of PFIs and SFIs from the gold bearing quartz vein we determined by *in vacuo* progressive ^40^Ar/^39^Ar dating in this study are 308.9 ± 6.8 and 298.0 ± 4.3, respectively (Fig. 5c), which are consistent with the modern atmospheric ^40^Ar/^36^Ar ratio, indicating that there is no significant excess ^40^Ar in either PFIs or SFIs. Therefore, we infer the ore-forming fluids of the Liaotun gold deposit to be mainly derived from meteoric waters transported by regional ore-controlling faults and/or basinal fluids derived by gravitational pressure.

The Ar-isotopic composition of fluids trapped in fluid inclusions carries a signature of the source of the fluid^32,35,40,41,48,49^. Previous studies have shown that various argon reservoirs are tapped during *in vacuo* crushing experiments, including PFIs, SFIs, microcracks, crystal defects and mineral interfaces^36,40,42,43,48–50^. In addition, atmospheric argon trapped in the stainless steel crusher may be released after intense crushing^36,48^. Based on the data point distribution on inverse isochron plot (Fig. 5), the gas release process can be grouped into two stages: mixed gases from SFIs and PFIs in the initial and medium steps; and dominantly PFIs in the final steps of the crushing procedure. The SFIs have higher ^39^Ar_K_, ^38^Ar_Cl_, ^37^Ar_Ca_ contents (Fig. 6) and a K/Ca ratio (Fig. 5b) than the PFIs, reflecting relatively higher potassium and chlorine contents dissolved in the SFIs. This may indicate that the source of the SFI-fluids had extensive water–rock interaction with the country rocks, resulting in much potassium dissolved during their migration, which is consistent with the ore deposit being hosted Triassic siltstone and mudstone that are rich in potassium-bearing minerals, e.g., mica, sericite, illite, kaolin-group minerals and K-feldspar^10,54^. The SFIs may therefore derive from the meteoric waters transported along regional faults and the high potassium in the SFIs could be related to Cl^-^ and/or HCl^-^ dissolved in the fluids. In contrast, the PFIs have lower contents of ^37^Ar_Ca_ and ^38^Ar_Cl_ and a lower K/Ca ratio (Figs. 5b, 6). This suggests that the ore-forming hydrothermal fluids had intensive water–rock reaction with calcium-rich, rather than potassium-rick rocks, and the potassium in the PFIs is probably related to HCO_3_^-^ and CO_2_^-^ dissolved in the ore-forming fluid^40^. Thus, our results show that the origin of the PFIs is likely in gravitational pressure derived basinal fluids, which migrated through carbonaceous rocks within the sedimentary basin.

### Significance of ages obtained by ^40^Ar/^39^Ar crushing dating

Fluid inclusion dating is a very important technique in modern studies of hydrothermal mineral deposits, as the trapped fossil fluid inclusions provide pivotal information on the geochemistry and geochronology of mineralization systems^55–58^. Critical to obtaining mineralisation ages, and one of the most challenging aspects of the geochronology of fluid inclusions is how to effectively distinguish and extract the gases from primary and secondary fluid inclusions, respectively^31,38–40,42,53,59^. We follow the definitions of Bodnar^60^, who indicated that fluid inclusions the form during and resulting from growth of the host crystal are considered primary. Secondary fluid inclusions form if a crystal fractures and fluid is trapped when the fracture heals. Secondary fluid inclusions thus postdate crystal growth.

As mentioned above, the SFIs in sample LQ19-1-2Qz are large (5–10 μm) and mainly occur along cross-cutting healed fractures, causing their fluids to be easily extracted during the initial crushing steps. In contrast, PFIs are generally smaller than SFIs, more isolated, and randomly distributed in host crystals. This means they likely need more impacts to crack them, but fluids from rare larger-volume PFIs may be liberated during early to middle crushing steps. Experiments testing crushing approaches show that as long as the crushing times are enough, gases from most of the > 1 μm fluid inclusions can be extracted effectively^39,42^. Meanwhile, many ^40^Ar/^39^Ar dating experiments have shown that quartz samples dated by the progressive crushing ^40^Ar/^39^Ar technique can provide good ages if they have abundant fluid inclusions with salinities generally higher than 8.0wt.% NaCl equivalent^31^. This suggest that the PFIs from this study with salinities higher than 9.9 wt% NaCl equivalent allow us to obtain a geologically meaningful age from the PFIs.

#### Significance of the ~ 167 Ma first plateau age

In the case of sample LT19-1-2Q, the liberated gas from stages 5–11 yields an inverse isochron age of 167.0 ± 1.9 Ma (Fig. 5c). Following the reasoning of Qiu and co-workers^31,36,40,49^ we infer that gas from the SFIs can be easily liberated during the early crushing steps by the *in vacuo* crushing method, due to their relatively large volume and distribution characteristics along healed cracks in their host crystals. Therefore, the inverse isochron age of the first segment can in most cases be interpreted as a separate, post-mineralisation fluid pulse recorded by SFIs^31,48,59^. However, in this study, the possibility that the Ar liberated in the first age segment was affected by release from both PFIs and SFIs with different proportions should be considered for the following reasons. First, as shown in Fig. 5c, the progressive crushing data points from the first step to the last step describe a clockwise sequence on ^36^Ar/^40^Ar *vs.* ^39^Ar/^40^Ar isotope correlation diagram. Specifically, in the crushing process, the data change from low to high ^39^Ar/^40^Ar ratios for the SFI correlation line and then to low ^39^Ar/^40^Ar along the PFI correlation line. This suggests that the trend of the ratios for steps 12–15 reflect and increasing proportion of gasses from PFI. Secondly, both ^40^Ar^*^ and ^39^Ar_K_ increase from very low contents in the first few steps to their peak in the intermediate steps, followed by a slow decline in the final steps (Fig. 6). This shows that, although in the middle stages the sample has not yet been fully crushed, most gas is then liberated, which supports the hypothesis that the gas compositions in the middle steps are mixtures of PFIs and SFIs. Finally, as shown in Fig. 1b, published isotopic ages indicating the metallogenic ages of the Carlin-type gold deposits in the north of the Golden Triangle are mainly concentrated at 150–130 Ma and 223–191 Ma^1–4,24^, while the magmatic activity in this region is concentrated at 96–77 Ma^18,30,61^. The first plateau age of *ca*. 167 Ma obtained here for crushing stages 5–11 is intermediate between the two groups of mineralisation ages. Therefore, we interpret this age as an upper age limit for an episode of late hydrothermal fluid activity after the formation of gold deposit and recorded by the SFIs in the quartz. We suggest that this age of *ca.* 167 Ma needs further study, and future Raman analysis of fluid inclusions coupled with quadrupole mass spectrometer analyses of the gases released during progressive crushing may provide new insights into the processes of releasing gases form fluid inclusions and thus enable a better understanding the geological significance of this age^31,42^.

#### Significance of the ~ 200 Ma second plateau age

With continued crushing, steps 16 through 33 form a flat age spectrum yielding a well-defined isochron with an age of 200.5 ± 1.9 Ma (Fig. 5c). This segment is interpreted as the contribution from the radiogenic (^40^Ar_R_) and trapped argon in the PFIs. The data points of the PFIs show a gradual progression along the correlation line toward the ^36^Ar/^40^Ar intercept as crushing proceeds (Fig. 5c), indicating an increasing non-radiogenic Ar component towards the end of the crushing experiment. Previous studies suggested the air released from the crusher becomes more dominant due to the very fine grain sizes and the high number (several hundreds) of pestle drops during late-stage crushing analyses (Table S1)^36,42,49,59^, but the possibility that some trapped Ar is released from the quartz lattice itself and dominates the non-radiogenic component cannot be completely eliminated.

Since the pyritized gold-bearing quartz vein in Liaotun is related to the main-metallogenic stage and its gold grade is as high as *ca.* 4 g/t Au, the age of PFIs determined in this study can be taken as the best estimate for the timing of Au mineralization, which is coeval with the metallogenic age of the main Carlin-type gold deposits in the central and southern part of the Youjiang ore concentration area in South China^1,2,4^. Combining this result with previous studies^1,2,4,7,19,44^, we suggest that the Liaotun gold deposit formed during the transition from collisional compression to extensional tectonics in the early Jurassic.

### Advantages of ^40^Ar/^39^Ar dating by progressive crushing

Fluid inclusions are usually trapped at multiple times during the existence of a crystal. They may be captured at various times during crystal growth, but also during subsequent fracturing and healing of the crystal^55^. The main drawback of conventional fluid extraction techniques applied to mineral separates, such as the Rb–Sr isochron method, is that they extract different generations of fluid inclusions simultaneously^42,62^. As a result, mixing of PFIs and SFIs will cause scatter of the data points or result in a meaningless mixed Rb–Sr isochron age if inclusions were formed over a relatively long time interval. In contrast, the ^40^Ar/^39^Ar progressive crushing technique of fluid inclusions has overcome this obstacle, and PFIs and SFIs in a sample can potentially be separated by progressive crushing owing to their various volume and distinctive distribution characteristics^36,39–42^. Furthermore, we have demonstrated that an age spectrum and isochron line can be obtained from just one sample by ^40^Ar/^39^Ar progressive crushing, and using the ^36^Ar/^40^Ar *vs* ^39^Ar/^40^Ar inverse isochron diagram, contamination of excess ^40^Ar, if present, can be eliminated by the initial ^40^Ar/^36^Ar ratio from the isochron^37,38^. Last but not least, the correlations of K, Ca, Cl and Ar isotopes derived from neutron irradiation have the potential to be used to obtain PFI and SFI ages^37,40,42,48^, and when combined with the initial ^40^Ar/^36^Ar ratio from the isochron, they also can be used to fingerprint the source and evolution history of the ore-forming fluids^31,32,40,43,62,63^.

## Methods

Heating-freezing experiments on fluid inclusions were performed in doubly polished thick sections of the gold-bearing quartz vein using a Linkam THMS 600 freezing/heating stage coupled to a BX51 Olympus polarizing microscope at Guilin University of Technology, China. The rate of heating and cooling were ~ 10 °C/min and were reduced to 2 °C/min near phase changes. The homogenization temperatures (T_h_) of aqueous fluid inclusions that homogenize to the liquid phase and the temperatures of ice-melting (T_m_) were measured. Homogenization temperatures are the minimum trapping temperatures of fluid inclusions, whereas ice-melting temperatures provide a measure of the fluid salinity^47^.

The quartz sample from the gold-bearing quartz vein was crushed with a jaw crusher and sieved to obtain a size fraction of 500-1000 µm. The sieve fraction was put in HNO_3_ to dissolve the carbonate fraction, after which the sample was purified using heavy liquid separation (quartz density: 2.64–2.66 g/cm^3^). Finally, the sample was hand-picked under a binocular microscope and cleaned in an ultrasonic bath with deionized water for 30 min. Samples were wrapped in aluminium foil and loaded into aluminium vessels together with standards. The flux monitor standards for *J-*value calculation were ZBH-2506, with an assumed age of 132.7 ± 0.5 Ma^64^. This standard was inserted between every two to four samples. The irradiation time Mianyang Research Reactor in China for irradiation WH01 was 40 h.

*In vacuo* crushing experiments were carried out in an in-house designed crushing apparatus, which was connected to a three-stage extraction line and connected with an ARGUS VI noble gas mass spectrometer in Ministry of Education Key Laboratory of Tectonics and Petroleum Resources, China University of Geosciences (Wuhan). The crusher consists of a stainless-steel tube (height = 160 mm, diameter = 14.0 mm) with a spherical curvature on the internal base, and a magnetic stainless-steel pestle (R = 13.8 mm, 222 g). The tube, welded with a DN40CF flange at the top, was connected to the purification line through a bellow. After loading a sample through the flange, the pestle was inserted into the tube. The pestle was moved to the bottom using a permanent strong magnet tool enveloped by a nonmagnetic sleeve. The pestle was lifted and dropped with a frequency of 2 Hz using an external electromagnet. The pestle was dropped in free fall from a height of 4–5 cm to crush the sample gently, and then the gases released from fractured fluid inclusions in the quartz sample were extracted. In order to maintain a sufficient level of argon available for analysis, the number of pestle drops per extraction step was increased through the experiment (Table S1). Cool blank analyses were carried out at the start and end of the experiment and between every five to eight steps of sample measurements for correcting the system blanks. These blanks were measured in a static state without the movement of the pestle rather than crushing the empty tube with the risk of liberating significant amounts of air trapped within the steel. The gases released were purified by a Zr/Al getter pump operated at room temperature and another Zr/Al pump operated at 400 °C for 400 s. Mass discrimination (0.99745–0.99749 per atomic mass unit) was monitored by frequent analysis of ^40^Ar/^36^Ar reference gas pipette aliquots. Correction factors for interfering argon isotopes derived from Ca and K isotopes were: (^39^Ar/^37^Ar)_Ca_ = 0.0006175, (^36^Ar/^37^Ar)_Ca_ = 0.002348, (^40^Ar/^39^Ar)_K_ = 0.002323 and (^38^Ar/^39^Ar)_K_ = 0.009419. The ^40^Ar/^39^Ar data were calculated and plotted using the ArArCALC software package of Koppers^65^. Detailed data and relevant parameters for ^40^Ar/^39^Ar progressive crushing experiments are listed in Supplementary Table S1. Age spectrum and inverse isochron of the sample is illustrated in Fig. 5. Both the plateau and inverse isochron age uncertainties are given at the 2σ level.

## Supplementary Information

Supplementary Table S1.

Supplementary Table S2.

## Data Availability

All data are reported in the Supplementary Information.
