# Supplementary material for: Progressive crushing 40Ar/39Ar dating of a gold-bearing quartz vein from the Liaotun Carlin-type gold deposit, Guangxi, southern China
Source: Sci Rep. 2022 Jul 27;12:12793. doi: 10.1038/s41598-022-17061-x (PMC9329373; doi:10.1038/s41598-022-17061-x)
Supplement: Supplementary file 2 — Supplementary Table S2. [file 41598_2022_17061_MOESM2_ESM.docx]

Table S2. Microthermometric data of two-phase aqueous inclusions from the typical deposits in the Dian-Qian-Gui region

|  |  | Fluid inclusion assemblage | |  | Microthermometric data | | |  |
| --- | --- | --- | --- | --- | --- | --- | --- | --- |
| Deposits | Stage | Generation | No. |  | T_h_ | T_m_ | Salinity | Reference |
| Liaotun | Main-stage quartz | Primary | 1 |  | 229.1 | -9.5 | 13.4 | This study |
|  |  |  | 1 |  | 245.3 | -8.3 | 12.0 |  |
|  |  |  | 1 |  | 206.5 | -7.3 | 10.9 |  |
|  |  |  | 1 |  | 180.5 | -6.5 | 9.9 |  |
|  |  |  | 1 |  | 208.4 | -7.7 | 11.3 |  |
|  |  |  | 1 |  | 235.6 | -8.1 | 11.8 |  |
|  |  |  | 1 |  | 193.5 | -6.7 | 10.1 |  |
|  |  |  | 1 |  | 198.5 | -6.9 | 10.4 |  |
|  |  |  | 1 |  | 215.4 | -7.3 | 10.9 |  |
|  |  | Secondary | 1 |  | 160.4 | -6.5 | 9.9 |  |
|  |  |  | 1 |  | 177.5 | -2.1 | 3.5 |  |
|  |  |  | 1 |  | 181.1 | -7.5 | 11.1 |  |
|  |  |  | 1 |  | 188.3 | -5.5 | 8.5 |  |
|  |  |  | 1 |  | 199.6 | -6.1 | 9.3 |  |
|  |  |  | 1 |  | 174.5 | -3.5 | 5.7 |  |
|  |  |  | 1 |  | 175.8 | -4.6 | 7.3 |  |
|  | Main-stage quartz | Primary/Secondary | 73 |  | 160–240 | –3.2 to –8.5 | 5.3–12.3 | 1 |
|  |  |  |  |  |  |  |  |  |
| Shuiyindong | Early-stage quartz | Primary | 14 |  | 218–231 | –3.5 to –4.3 | 5.7–6.9 | 2 |
|  |  | Secondary | 18 |  | 194–229 | –2.3 to –3.3 | 3.9–5.4 |  |
|  | Late-stage calcite | Primary | 28 |  | 83–200 | –0.1 to –1.0 | 0.2–1.7 | 3 |
|  | Main-stage quartz | Primary |  |  | 149–273 | –0.2 to –5.8 | 0.3–9.0 | 4 |
|  | Early-stage quartz | Primary | 46 |  | 183–314 |  | 3.9–11.1 | 5 |
|  | Main-stage quartz | Primary | 64 |  | 166–271 |  | 1.9–13.7 |  |
|  | Late-stage calcite | Primary | 20 |  | 152–193 |  | 3.9–8.7 |  |
|  |  |  |  |  |  |  |  |  |
| Yata | Early-stage quartz | Primary | 23 |  | 190–258 | –3.0 to –4.1 | 5.0–6.6 | 2 |
|  | Main-stage quartz | Secondary | 22 |  | 165–230 | –2.1 to –3.3 | 3.6–5.4 |  |
|  |  |  |  |  |  |  |  |  |
|  | Late-stage quartz and stibnite | Primary in stibnite | 12 |  | 178–212 | –1.7 to –3.0 | 2.9–5.0 | 6 |
|  |  | Secondary in quartz | 23 |  | 151–261 | –1.2 to –4.5 | 2.1–7.2 | 2 |
|  | Main-stage quartz | Primary |  |  | 125–195 | –0.5 to –6.1 | 0.9–9.3 | 4 |
|  | Early-stage quartz | Primary | 32 |  | 171–241 |  | 3.9–11.1 | 5 |
|  | Main-stage quartz | Primary | 26 |  | 158–189 |  | 0.9–9.3 |  |
|  | Late-stage calcite | Primary | 15 |  | 131–176 |  | 2.9–6.6 |  |
|  |  |  |  |  |  |  |  |  |
| Lannigou | Early-stage quartz | Primary | 28 |  | 207–278 | –2.5 to –3.9 | 4.2–6.5 | 7 |
|  | Main-stage quartz | Secondary | 44 |  | 180–228 | –2.3 to –3.2 | 3.9–5.3 |  |
|  | Late-stage quartz and calcite | Secondary in quartz | 26 |  | 117–166 | –0.1 to –3.2 | 0.2–5.3 |  |
|  |  | Secondary in calcite | 25 |  | 116–207 | 0.0 to –3.9 | 0.0–6.3 | 8 |
|  | Late-stage quartz | Primary |  |  | 137–149 | –0.7 to –4.7 | 1.2–7.4 | 4 |
|  |  |  |  |  |  |  |  |  |
| Taipingdong | Early-stage quartz | Primary | 84 |  | 180–281 | –0.6 to –5.2 | 1.1–8.1 | 3 |
|  | Main-stage quartz | Primary | 170 |  | 170–295 | –0.2 to –4.7 | 0.4–7.4 |  |
|  | Late-stage calcite | Primary | 40 |  | 97–172 | –0.1 to –3.5 | 0.2–5.7 |  |
|  | Main-stage quartz | Primary |  |  | 159–315 | –0.6 to –5.2 | 1.1–7.1 | 4 |
|  |  |  |  |  |  |  |  |  |
| Zimudang | Main-stage quartz | Primary | 95 |  | 170–273 | –0.2 to –4.7 | 0.4–7.5 | 3 |
|  | Late-stage calcite | Primary | 73 |  | 88–209 | –0.1 to –3.5 | 0.2–5.7 |  |
|  |  |  |  |  |  |  |  |  |
| Bojitian | Late-stage calcite | Primary | 45 |  | 80–198 | –0.5 to –6.5 | 0.9–7.5 | 3 |
|  |  |  |  |  |  |  |  |  |
| Nibao | Early-stage quartz | Primary | 46 |  | 194–301 | –0.4 to –4.8 | 0.7–7.6 | 9 |
|  | Main-stage quartz | Primary | 58 |  | 125–278 | –0.3 to –3.9 | 0.5–6.3 |  |
|  | Late-stage calcite | Primary | 11 |  | 133–197 | –0.3 to –4.7 | 0.5–7.5 |  |
|  | Late-stage fluorite | Primary | 53 |  | 102–264 | –0.1 to –2.7 | 0.2–4.5 |  |
|  | Early-stage quartz | Primary | 39 |  | 159–271 |  | 3.9–13.7 | 5 |
|  | Main-stage quartz | Primary | 16 |  | 165–228 |  | 2.7–7.6 |  |
|  | Late-stage calcite | Primary | 17 |  | 137–195 |  | 3.6–8.7 |  |
|  | Late-stage quartz | Primary |  |  | 157–226 | –2.3 to –3.4 | 3.9–5.6 | 4 |
| Mingshan | Main-stage quartz | Primary | 85 |  | 122–358 | –0.3 to –6.2 | 0.5–9.5 | 10 |
| All values are in °C for temperature and wt % NaCl equiv for salinity | | |  |  |  |  |  |  |
| Abbreviations: Th = homogenization temperature, Tm = melting temperature of ice | | | | | |  |  |  |

References

1 Li, Y. Q. Geology, Geochemistry and Genesis of the Liaotun Gold Depisit, Bama county, Guangxi, China. *Guilin University of Technology* (2016).

2 Su, W. C. *et al.* Sediment-Hosted Gold Deposits in Guizhou, China: Products of Wall-Rock Sulfidation by Deep Crustal Fluids. *Econ Geol*. **104**, 73-93 (2009).

3 Peng, Y. W. *et al.* Ore-forming process of the Huijiabao gold district, southwestern Guizhou Province, China: Evidence from fluid inclusions and stable isotopes. *J Asian Earth Sci*. **93**, 89-101 (2014).

4 Wu, S. Y. *et al.* Ore-controlling structure types and characteristics of ore-forming fluid of the Carlin-type gold orefield in southwestern Guizhou, China. *Acta Petrologica Sinica*. **32**, 2407-2424 (2016).

5 Wu, S. Y. The Study of Tectonic-Magmatic-Hydrothermal Metallogenic Model of Carlin-type Gold Deposit in Southwestern Guizhou Province, China (in Chinese). *Ph.D. Dissertation. Beijing: China University of Geosciences*, 1-230 (2019).

6 Su, W. C. *et al.* Carlin-Type Gold Deposits in the Dian-Qian-Gui “Golden Triangle” of Southwest China. *Rev Mineral Geochem*. **20**, 157-185 (2018).

7 Su, W. C. The hydrothermal fluid geochemistry of the Carlin-type gold deposits in Southwestern Yangtze Craton, China (in Chinese). *Ph.D. Dissertation. Guiyang: Institute of Geochemistry, Chinese Academy of Sciences*, 1-127 (2002).

8 Zhang, X.-C., Spiro, B., Halls, C., Stanley, C. J. & Yang, K.-Y. Sediment-Hosted Disseminated Gold Deposits in Southwest Guizhou, PRC: Their Geological Setting and Origin in Relation to Mineralogical, Fluid Inclusion, and Stable-Isotope Characteristics. *Int Geol Rev*. **45**, 407-470 (2003).

9 Xie, X. Y. *et al.* Fluid inclusion and stable isotope geochemistry study of the Nibao gold deposit, Guizhou and insights into ore genesis. *Acta Petrologica Sinica*. **32**, 3360-3376 (2016).

10 Chen, M. H. Geological and ore-forming fluid charateristics of the Mingshan gold deposit in Western Guangxi. *Miner Depos*. **29**, 913-914 (2010).
